# Time-Resolved Developmental and Transcriptomic Profiling of Potato Stolon Initiation from Basal Axillary Buds Following Soil Covering

**DOI:** 10.3390/ijms27177633

**Published:** 2026-08-26

**Authors:** Kaiyuan Zhao, Baozhen Jiao, Yuan Sun, Tingting Wang, Yongchao Wu, Binquan Huang

**Affiliations:** 1 School of Agriculture, Yunnan University, Kunming 650500, China; 2 Institute of Vegetables, Anhui Academy of Agricultural Sciences, Hefei 230001, China

**Keywords:** potato, stolon initiation, basal axillary bud, soil covering, time-series transcriptomics, Mfuzz clustering

## Abstract

Potato stolon research has emphasized stolon-tip tuberization, leaving the transition of basal axillary buds into subterranean stolons poorly characterized. Using cv. Désirée, we characterized this transition at IS0 immediately before soil covering and at 5, 7, 9, and 11 d thereafter (IS5, IS7, IS9, and IS11) by morphological, histological, and transcriptomic analyses. At IS5, external morphology changed little, although internal tissue reorganization was evident. During IS7–IS9, buds progressively reoriented, enlarged basally, and established a subterranean lateral axis. By IS11, white stolons had formed and entered post-initiation elongation without visible stolon-tip swelling. PCA separated IS0 and IS11 from the intermediate stages, and adjacent-stage differential expression identified the largest DEG sets in IS0–IS5 and IS9–IS11. Direction-resolved GO and KEGG enrichment distinguished successive transitions: early oxidative/defense and phenylpropanoid-related categories; intermediate changes in transport, photosynthesis, redox metabolism, and cytoskeletal organization; and later enrichment of translation-, proteasome-, and cell-division-related categories. Mfuzz clustering provided a complementary overview of broad temporal trajectories, including IS5-associated C6, distinct C3 and C5 patterns during initiation, and IS11-associated C2. RT-qPCR profiles of *StBRC1b*, *StWUS*, *StWOX5*, *StCCD8*, *StSP6A*, and *StSP3D* were broadly consistent with the RNA-Seq trends. These results establish a time-resolved framework for soil-covering-induced stolon initiation and subsequent elongation.

## 1. Introduction

Potato (*Solanum tuberosum* L.) stolons are specialized subterranean lateral stems that arise from axillary buds at basal or underground nodes and provide the developmental foundation for subsequent tuber formation. Their development comprises successive but biologically distinct processes. Stolon initiation denotes the emergence and progressive establishment of a subterranean lateral axis from an axillary bud, whereas stolon elongation refers to axial growth after that lateral axis has formed. Stolon-tip swelling and tuberization occur later and involve radial expansion, altered patterns of cell division and expansion, storage-reserve accumulation, and differentiation of tuber tissues [1,2,3,4]. These stages must be distinguished because evidence from tuber induction, stolon-tip swelling, or tuber growth cannot be assumed to explain the preceding bud-to-stolon transition.

Current molecular understanding of potato subterranean development centers on photoperiod-dependent tuber induction and the subsequent stolon-to-tuber transition. The FT-like protein StSP6A functions as a mobile tuber-inducing signal produced in source leaves and transported to subterranean tissues [5]. StSP6A interacts with 14-3-3 and FD-like proteins in a tuberigen complex [6], and its interaction with SWEET11 links tuberigen signaling to source–sink regulation [7]. At the transcriptional level, *StCOL1* activates the FT-like repressor gene *StSP5G*, thereby repressing *StSP6A* expression [8], whereas the StBEL5 transcription factor regulates targets that include *StSP6A* [9]. The FT-like proteins StSP3D and StFTL1 also act as long-distance signals associated with potato storage-organ formation [10]. This framework is central to understanding tuber induction and later subterranean development, but it does not resolve how an initially appressed basal axillary bud first establishes stolon morphology.

Stolons originate from lateral meristematic tissues and are therefore developmentally related to, but distinct from, conventional shoot branches. In potato, the TCP transcription factor StBRC1b contributes to the spatial regulation of subterranean development and tuberization [11]. Functional analysis of the *StCCD8* gene has linked strigolactone biosynthesis to branching, stolon architecture, and tuber development [12,13]. Independent loss-of-function and overexpression analyses of *StLAX5*, which encodes an AUX/LAX-family auxin influx carrier, showed that *StLAX5*-mediated auxin influx contributes to potato stolon primordium formation [14]. More broadly, axillary bud activity and sustained lateral-axis growth integrate auxin, cytokinins, strigolactones, abscisic acid, gibberellins, carbon availability, and environmental inputs [15,16].

Studies of other stolon-forming and branching systems provide additional developmental context. In woodland strawberry, environmental and endogenous inputs influence whether axillary buds remain quiescent or develop into branch crowns or stolons [17], and genetic analysis has shown that light, gibberellin, and ZFP6 regulate axillary-meristem maturation and stolon fate [18]. Transcriptomic analysis of petunia axillary buds has similarly implicated ABA signaling and carbon supply in differential bud outgrowth [19]. WUSCHEL-related homeobox proteins are broadly associated with meristem organization and stem-cell maintenance [20], and recent work has defined transcriptional and chromatin-associated functions of WOX5 in the *Arabidopsis* root stem-cell organizer [21]. These studies provide a comparative basis for examining meristem-related candidates in potato, including *StWUS* and *StWOX5*.

Recent transcriptomic studies have expanded the molecular characterization of potato subterranean development. Comparative transcriptome and co-expression analyses have examined leaves, stems, unswollen stolons, swollen stolon regions, and early tuber tissues during the stolon-to-tuber transition [22], while leaf and stolon transcriptomes have been used to investigate phytochrome F and photoperiod-dependent tuberization [23]. Single-cell profiling has compared cell states in the potato shoot apex and established stolon tips [24], and a separate time course examined hormone- and sugar-associated responses during sucrose-induced stolon initiation from stem nodes [25]. However, these studies differ in tissue resolution, induction conditions, or developmental endpoints and do not provide a continuous morphological, histological, and transcriptomic account of a soil-covered basal axillary bud progressing from its initial state through visible stolon establishment to post-initiation elongation.

In this study, potato cv. Désirée was subjected to soil covering, and basal axillary buds and associated developing tissues were examined at IS0, IS5, IS7, IS9, and IS11. Morphological and histological staging was integrated with bulk RNA-Seq, principal component analysis, differential expression between adjacent stages, separate GO and KEGG enrichment analyses of up- and downregulated DEGs, Mfuzz temporal clustering, representative-gene expression profiling, and RT-qPCR. Adjacent-stage enrichment characterized individual developmental transitions, whereas Mfuzz clustering summarized broader temporal trajectories. We aimed to establish a stage-resolved developmental and transcriptional framework for soil-covering-induced stolon initiation and subsequent elongation.

## 2. Results

### 2.1. Morphological and Histological Staging of Basal Axillary Bud Development Following Soil Covering

**Figure 1:**
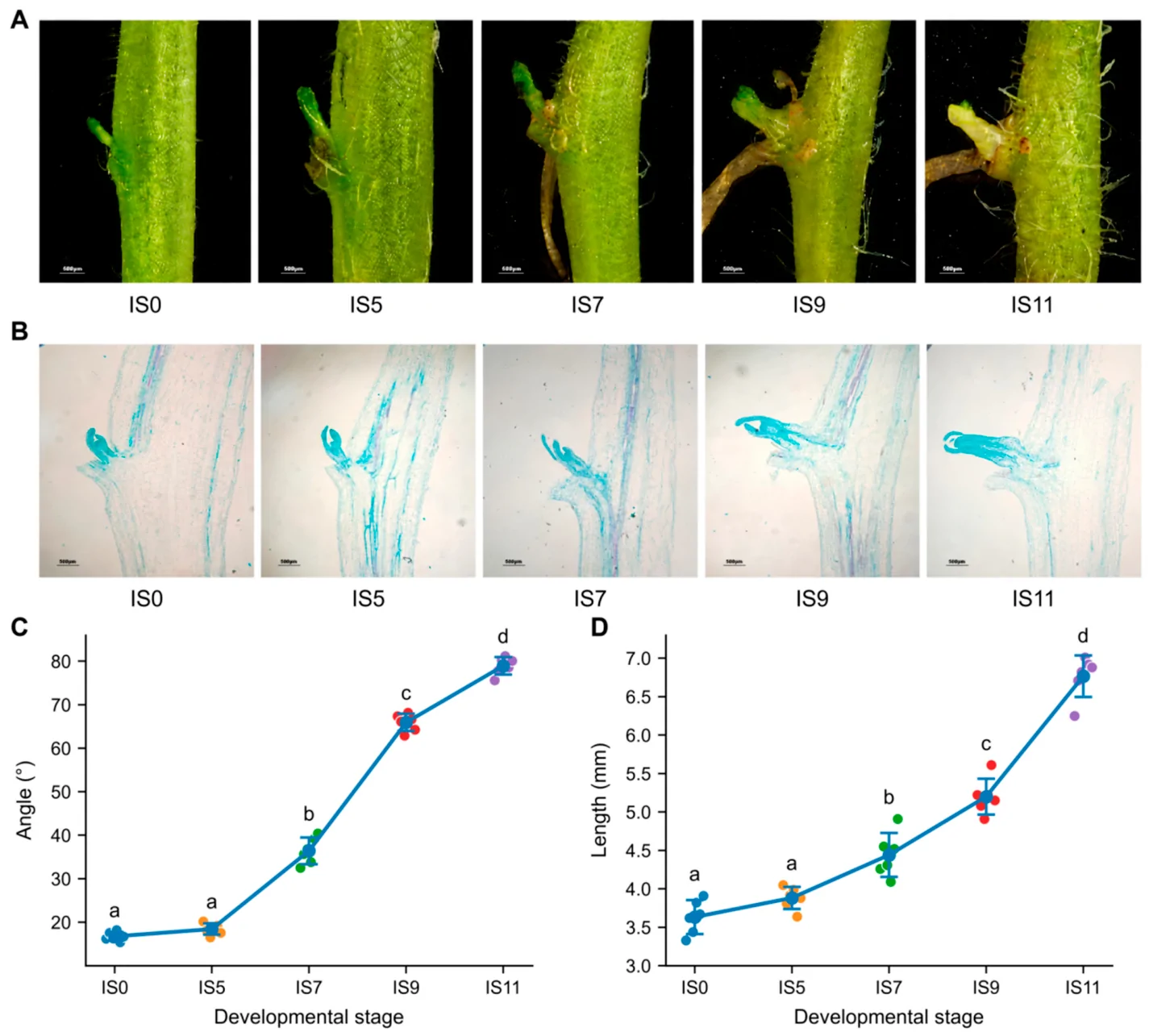
Morphological and histological staging of basal axillary bud development following soil covering. (**A**) Representative external morphology of basal axillary buds and developing subterranean stolons at IS0, IS5, IS7, IS9, and IS11. (**B**) Representative longitudinal sections showing changes in the bud apical region, the bud–main stem junction, and the developing lateral axis. (**C**) Angle between the basal axillary bud or developing stolon axis and the main stem. (**D**) Length of the basal axillary bud or developing stolon. IS5 represents the early internal-response subphase within the IS5–IS9 soil-covering-induced stolon-initiation window; IS7–IS9 represents morphologically evident stolon initiation and subterranean lateral-axis establishment; IS11 represents post-initiation elongation of an established white stolon. Scale bars in (**A**,**B**) = 500 μm. Quantitative data are presented as the mean ± SD (*n* = 6 independent plants per stage). Different lowercase letters indicate significant differences among stages determined by one-way ANOVA followed by Tukey’s multiple-comparison test at *p* < 0.05.

To characterize the developmental progression of potato basal axillary buds following soil covering, samples were collected immediately before treatment and at 5, 7, 9, and 11 d after soil covering. These stages were designated IS0, IS5, IS7, IS9, and IS11, respectively. Stage classification was based on external morphology, bud orientation, the angle between the bud or developing stolon and the main stem, bud or stolon length, and longitudinal histological observations (Figure 1). Representative tissue-cultured plantlets used for propagation are shown in Supplementary Figure S1.

At IS0, the basal axillary bud was small and closely appressed to the main stem, with no evident lateral extension (Figure 1A). At IS5, the bud remained appressed and exhibited only limited external change. At IS7, the bud–stem angle increased and the bud began to grow laterally away from the main stem. At IS9, lateral extension became pronounced, the bud base enlarged, and the developing axis acquired the morphology of an initiating subterranean stolon. At IS11, a slender white subterranean stolon had formed and showed marked longitudinal elongation, without visible stolon-tip swelling or tuberization.

Longitudinal sections showed corresponding changes in tissue organization (Figure 1B). At IS0, the compact axillary bud remained closely connected to the main stem. Although external morphology changed little at IS5, subtle tissue reorganization was evident in the bud apical region and at the bud–main stem junction. At IS7, axial extension and structural changes at the basal junction became more evident. At IS9, an elongated lateral axis and enlarged basal region were clearly visible. At IS11, the sections showed a well-defined, elongated axial structure consistent with an established subterranean stolon.

Quantitative measurements supported the morphological staging. The angle between the bud and the main stem was 16.73 ± 1.02° at IS0 and 18.42 ± 1.30° at IS5, with no significant difference between these stages. The angle then increased to 36.40 ± 3.05° at IS7, 65.92 ± 1.98° at IS9, and 78.93 ± 2.04° at IS11, with significant differences among IS7, IS9, and IS11 (Figure 1C). The length of the bud or developing stolon was 3.63 ± 0.22 mm at IS0 and 3.88 ± 0.14 mm at IS5, with no significant difference between these stages. It then increased to 4.44 ± 0.29 mm at IS7, 5.20 ± 0.23 mm at IS9, and 6.77 ± 0.27 mm at IS11, with each stage differing significantly from the preceding stage (Figure 1D).

On the basis of the combined external morphological, histological, and quantitative evidence, IS5 was classified as an early internal-response subphase within the broader IS5–IS9 initiation window. IS7–IS9 represented morphologically evident stolon initiation and establishment of the subterranean lateral axis. IS11 represented post-initiation stolon elongation after the white lateral axis had formed.

### 2.2. Global Transcriptomic Profiling and Differential Expression Across Developmental Stages

RNA-Seq was performed for IS0, IS5, IS7, IS9, and IS11 using three biological replicates per stage, yielding 15 libraries. Each library generated 19.91–22.14 million clean read pairs, corresponding to 5.97–6.64 Gb of clean sequence data. Q20 and Q30 values ranged from 97.81% to 98.31% and from 92.09% to 93.85%, respectively; complete quality-control statistics are provided in Supplementary Table S1.

**Figure 2:**
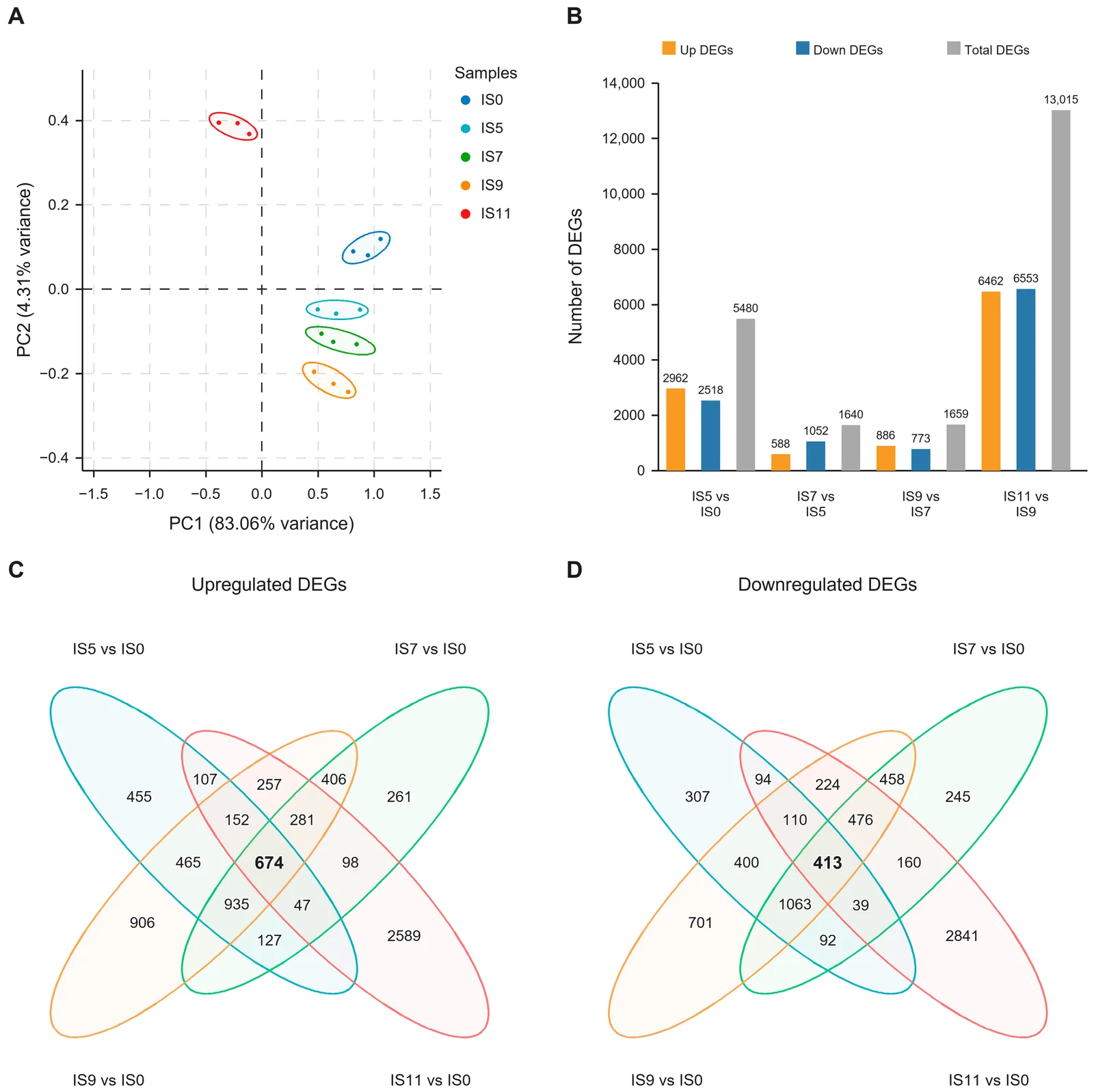
Global transcriptomic relationships and differential expression across developmental stages following soil covering. (**A**) Principal component analysis of 15 RNA-Seq libraries representing five developmental stages, with three biological replicates per stage. Each point represents one biological replicate; colors denote developmental stages. PC1 and PC2 explained 83.06% and 4.31% of the total variance, respectively. (**B**) Numbers of upregulated, downregulated, and total DEGs in the adjacent-stage comparisons IS5 vs. IS0, IS7 vs. IS5, IS9 vs. IS7, and IS11 vs. IS9. (**C**,**D**) Four-set Venn diagrams showing upregulated (**C**) and downregulated (**D**) DEGs in the IS0-reference comparisons IS5 vs. IS0, IS7 vs. IS0, IS9 vs. IS0, and IS11 vs. IS0. The four-way central intersections contain 674 commonly upregulated and 413 commonly downregulated genes. The regions shared by IS5, IS7, and IS9 but excluding IS11 contain 935 upregulated and 1063 downregulated genes. DEG identification criteria are described in Section 4.4.

Principal component analysis of the variance-stabilized expression matrix showed close clustering of the three biological replicates at each stage (Figure 2A). PC1 and PC2 explained 83.06% and 4.31% of the total variance, respectively. IS0 was separated from all post-covering stages. IS5, IS7, and IS9 occupied neighboring positions in PCA space but remained separated by stage, whereas IS11 formed a distinct group, consistent with the transition from the initiation window to post-initiation elongation.

Adjacent-stage differential expression analysis identified the largest numbers of DEGs at the beginning and end of the series (Figure 2B). IS5 versus IS0 contained 2962 upregulated and 2518 downregulated genes (5480 total DEGs); IS7 versus IS5 contained 588 upregulated and 1052 downregulated genes (1640 total); IS9 versus IS7 contained 886 upregulated and 773 downregulated genes (1659 total); and IS11 versus IS9 contained 6462 upregulated and 6553 downregulated genes (13,015 total). Thus, the IS0–IS5 early response and the IS9–IS11 transition to elongation involved more DEGs than the two intervening transitions.

The adjacent-stage comparisons in Figure 2B were distinct from the four IS0-reference comparisons in Figure 2C,D. Across IS5 vs. IS0, IS7 vs. IS0, and IS9 vs. IS0, 1609 genes were consistently upregulated and 1476 were consistently downregulated. Adding IS11 vs. IS0 distinguished responses maintained through elongation from those confined to the initiation window: 674 upregulated and 413 downregulated genes were shared across all four comparisons, whereas 935 upregulated and 1063 downregulated genes were shared by IS5, IS7, and IS9 but not IS11. The remaining intersections represented stage-restricted or partially shared responses.

The transcriptomic data therefore distinguished an IS5 early internal-response subphase, more gradual changes during morphologically evident initiation at IS7–IS9, and a pronounced transition accompanying post-initiation elongation at IS11.

### 2.3. Transition-Specific Functional Enrichment of Adjacent-Stage DEGs

**Figure 3:**
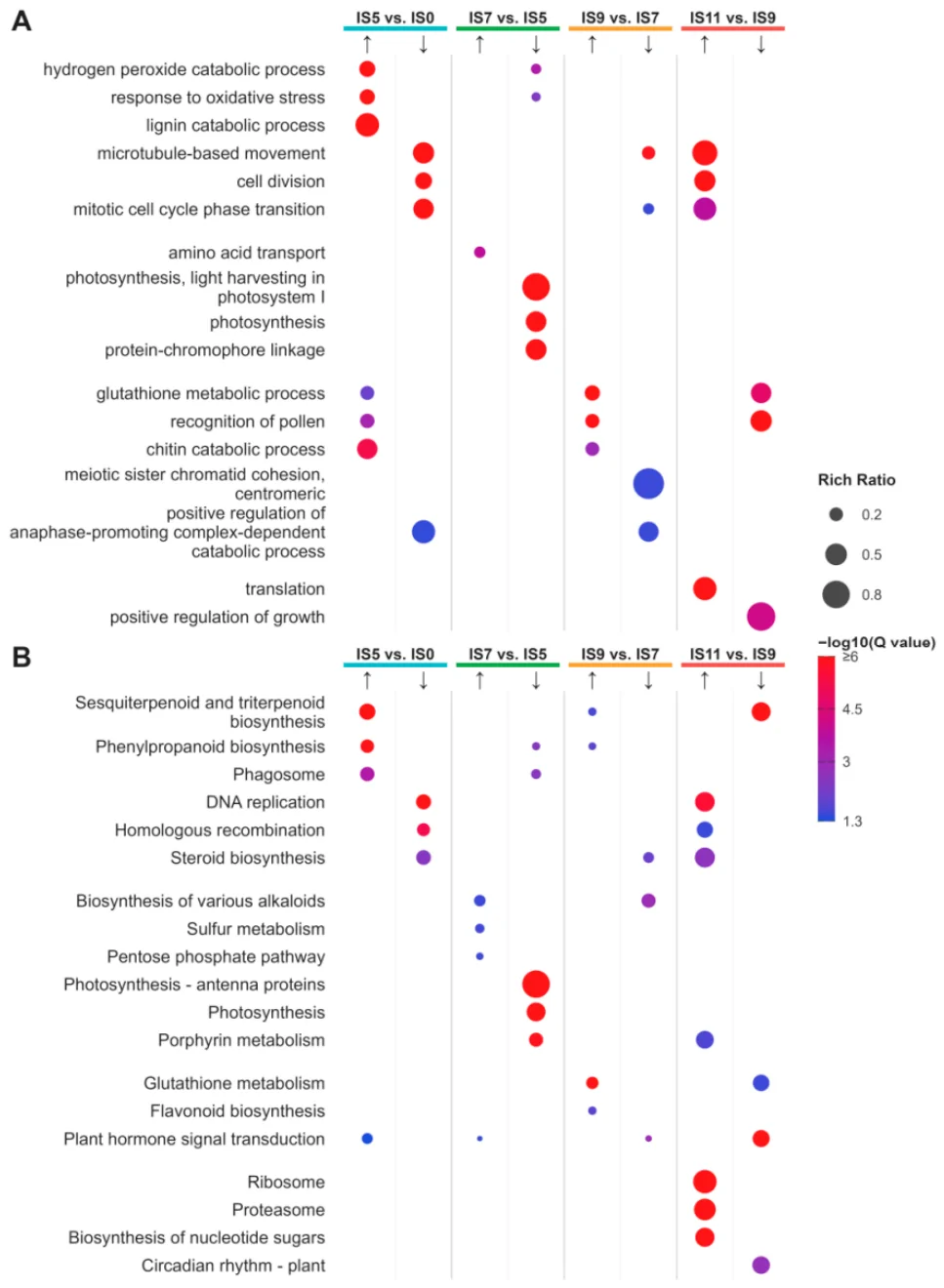
Direction-resolved functional enrichment of DEGs across adjacent developmental transitions following soil covering. (**A**) GO Biological Process terms and (**B**) KEGG pathways enriched among genes upregulated (↑) or downregulated (↓) at the later stage in IS5 vs. IS0, IS7 vs. IS5, IS9 vs. IS7, and IS11 vs. IS9. Benjamini–Hochberg-adjusted *Q* values < 0.05 were considered significant. Within each comparison–direction combination, significant categories were ranked by ascending *Q* value, descending Rich Ratio, and ascending stable identifier; up to three categories were selected. The union of the selected categories defined the matrix rows. For each selected category, all significant occurrences across the eight comparison–direction combinations are shown, and a blank cell indicates no significant enrichment in that combination. Bubble area represents Rich Ratio (*k*/*K*), where *k* is the number of direction-specific DEGs assigned to the category and *K* is the number of background genes assigned to that category. Bubble color represents −log_10_(*Q* value); values > 6 were capped at 6 for display. The colored annotation strips identify the four developmental transitions and do not encode significance or expression direction. Complete results are provided in Supplementary Tables S3 and S4.

To relate functional annotations directly to successive developmental transitions, upregulated and downregulated DEGs from each adjacent-stage comparison were analyzed separately for Gene Ontology Biological Process (GO BP) and KEGG pathway over-representation (Figure 3; Supplementary Tables S3 and S4). These analyses identify categories overrepresented within each directional DEG set but do not measure pathway activity.

In IS5 versus IS0, upregulated DEGs were enriched for hydrogen peroxide catabolic process, response to oxidative stress, and lignin catabolic process, whereas downregulated DEGs were enriched for microtubule-based movement, cell division, and mitotic cell-cycle phase transition (Figure 3A). KEGG pathways enriched among upregulated DEGs included sesquiterpenoid and triterpenoid biosynthesis, phenylpropanoid biosynthesis, and phagosome; downregulated DEGs were enriched for DNA replication, homologous recombination, and steroid biosynthesis (Figure 3B). The IS5 transition was therefore characterized by enrichment of categories related to oxidative stress and defense among upregulated genes and by enrichment of cell-cycle and DNA-replication categories among downregulated genes.

In IS7 versus IS5, amino acid transport was the only significant GO BP term among upregulated DEGs. Downregulated DEGs were enriched for photosynthesis, light harvesting in photosystem I, and protein–chromophore linkage. KEGG pathways enriched among upregulated DEGs included biosynthesis of various alkaloids, sulfur metabolism, and the pentose phosphate pathway, whereas downregulated DEGs were enriched for photosynthesis–antenna proteins, photosynthesis, and porphyrin metabolism. The transition from the IS5 internal response to visible lateral-axis emergence was therefore accompanied by changes in transport and intermediary metabolism and by downregulation of photosynthesis-related genes.

In IS9 versus IS7, upregulated DEGs were enriched for glutathione metabolic process, recognition of pollen, and chitin catabolic process; enriched KEGG pathways included glutathione metabolism, flavonoid biosynthesis, and phenylpropanoid biosynthesis. Downregulated DEGs were enriched for microtubule-based movement and chromosome- or cell-cycle-related terms, with KEGG enrichment in biosynthesis of various alkaloids, plant hormone signal transduction, and steroid biosynthesis. Thus, this transition combined redox and specialized-metabolism enrichment with downregulation of cytoskeletal, cell-cycle, and hormone-signaling functions.

In IS11 versus IS9, upregulated DEGs were enriched for translation, microtubule-based movement, and cell division, as well as the KEGG pathways ribosome, proteasome, and biosynthesis of nucleotide sugars. Downregulated DEGs were enriched for recognition of pollen, glutathione metabolic process, and positive regulation of growth; KEGG enrichment included plant hormone signal transduction, sesquiterpenoid and triterpenoid biosynthesis, and circadian rhythm–plant. These changes marked the transition from morphologically evident initiation to active elongation of the established stolon.

The four adjacent-stage comparisons showed distinct direction-specific enrichment profiles. Complete tested GO BP and KEGG results, including categories not displayed in Figure 3, are provided in Supplementary Tables S3 and S4.

### 2.4. Temporal Clustering Identifies Broad Expression Trajectories

**Figure 4:**
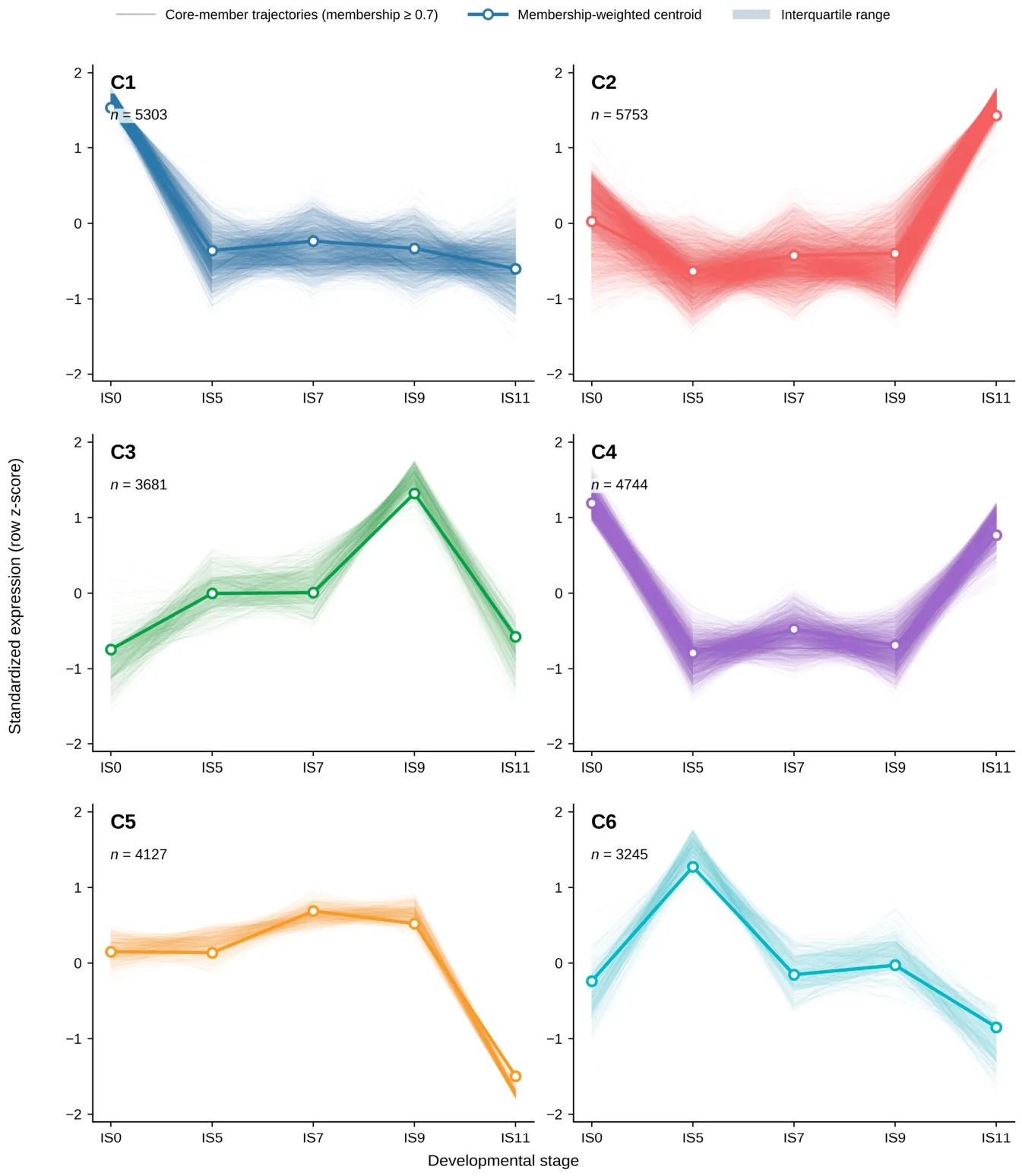
Broad temporal expression trajectories identified across developmental stages following soil covering. Genes were grouped into six Mfuzz soft clusters: C1, 5303 genes; C2, 5753 genes; C3, 3681 genes; C4, 4744 genes; C5, 4127 genes; and C6, 3245 genes. Expression values were calculated from stage-mean TPM values, transformed using log_2_(mean TPM + 1), and standardized by gene as row z-scores. The horizontal axis shows IS0, IS5, IS7, IS9, and IS11; the vertical axis shows standardized expression. Light-colored curves show high-membership trajectories (membership ≥ 0.7), the bold curve shows the membership-weighted centroid, and the shaded region indicates the interquartile range. The displayed *n* value denotes all genes assigned to each cluster. C3 and C5 remain separate clusters despite their joint use in one enrichment gene set.

Mfuzz soft clustering summarized broad expression trajectories across IS0, IS5, IS7, IS9, and IS11 (Figure 4). The six-cluster solution was treated as descriptive rather than as a uniquely optimized partition of developmental states. C1, C2, C3, C4, C5, and C6 contained 5303, 5753, 3681, 4744, 4127, and 3245 assigned genes, respectively. Membership values quantified the degree of association with each cluster; genes with membership ≥ 0.7 were used to display high-membership trajectories, whereas the reported *n* values denote all assigned genes. Complete cluster assignments and membership values are provided in Supplementary Table S5.

C1 showed its highest expression at IS0, declined at IS5, and remained below the IS0 level through IS11. C4 was also high at IS0 and declined at IS5, remained comparatively low through IS9, and increased again at IS11. Because both trajectories spanned multiple developmental transitions, they were not assigned to a single stage. Their enrichment profiles are shown in Supplementary Figure S3.

C6 reached its highest mean expression at IS5 and declined thereafter, corresponding to the early internal-response subphase. C3 and C5 remained separate clusters: C3 increased from IS0 to IS5, remained near that level at IS7, reached its maximum at IS9, and declined at IS11; C5 increased mainly at IS7, remained relatively high at IS9, and decreased sharply at IS11. C2 remained below its IS0 level during IS5–IS9 and increased markedly at IS11, corresponding to post-initiation elongation.

Cluster-based enrichment was used to annotate these broad trajectories (Supplementary Figures S2 and S3). Genes assigned to C6 were enriched in environmental and endogenous response, photosynthesis-related, lipid-metabolism, and cell-wall-associated categories. For enrichment only, genes assigned separately to C3 and C5 were pooled; this combined set was enriched in signal transduction, glutathione metabolism, fatty acid catabolism, protein processing, and vesicular transport. Genes assigned to C2 were enriched in translation, protein turnover, central carbon metabolism, protein processing, and intracellular transport. C1 showed enrichment in RNA modification, photosynthesis, and chloroplast organization, whereas C4 showed prominent DNA repair, DNA replication, RNA modification, and microtubule-related annotations. Complete GO BP and KEGG results for C6, C3+C5, and C2 are provided in Supplementary Tables S6 and S7; C1 and C4 are summarized in Supplementary Figure S3.

### 2.5. Representative Gene Expression Patterns Within Selected Temporal Clusters

**Figure 5:**
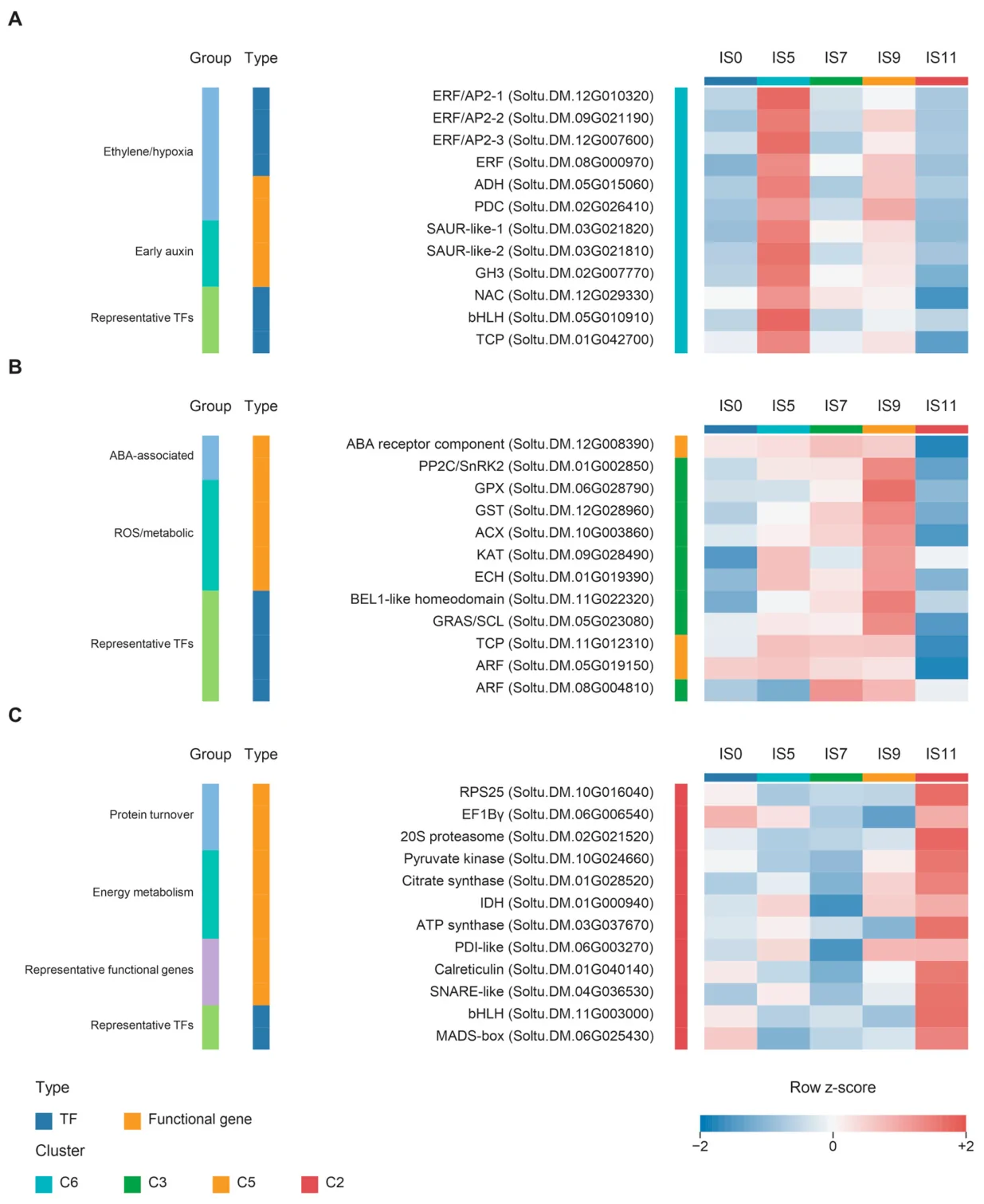
Temporal expression patterns of representative genes assigned to selected Mfuzz clusters. (**A**) Representative genes assigned to C6 and showing preferential expression at IS5. (**B**) Representative genes assigned separately to C3 and C5 and showing increased expression during IS7 and/or IS9; C3 and C5 are shown together only for visualization and remain separate Mfuzz clusters. (**C**) Representative genes assigned to C2 and showing preferential expression at IS11. Genes were selected according to cluster assignment, temporal trajectory, membership value, functional annotation, correspondence with enrichment results, and nonredundant functional coverage. Gene annotations and DM v6.1 identifiers are shown beside the heatmaps. Expression values were calculated from stage-mean TPM values, transformed using log_2_(mean TPM + 1), and standardized separately for each gene as row z-scores. Red and blue indicate expression above and below the gene-specific mean, respectively; colors therefore show temporal variation within each gene rather than absolute expression differences among genes. Annotation bars indicate functional groups, gene types, and cluster assignment. The displayed genes include both core (membership ≥ 0.7) and non-core cluster members.

Representative genes were selected for visualization on the basis of cluster assignment, temporal trajectory, membership value, functional annotation, correspondence with the cluster-enrichment results, and nonredundant functional coverage (Figure 5; Supplementary Table S9). The set included both core (membership ≥ 0.7) and non-core cluster members; therefore, these genes are described as representative rather than uniformly as core members.

Within C6, AP2/ERF, NAC, bHLH, and TCP transcription factors, together with genes annotated as ADH, PDC, SAUR-like, and GH3, showed transient increases at IS5 followed by lower expression at subsequent stages (Figure 5A). These profiles corresponded to the broad IS5 trajectory of C6.

C3 and C5 are displayed together in Figure 5B for comparison but remain distinct clusters. Among the C3 representatives, two BEL1-like homeodomain transcription factors, GPX, GST, ACX, KAT, ECH, GRAS/SCL, and an ARF gene increased at IS7 and/or IS9. Most reached their maximum at IS9, whereas Soltu.DM.08G004810 reached its maximum at IS7. The representative TCP and ARF genes assigned to C5 maintained comparatively high expression during IS7–IS9 and declined at IS11.

Representative C2 genes—including RPS25, EF1Bγ, a 20S proteasome component, pyruvate kinase, citrate synthase, IDH, an ATP synthase component, PDI-like, calreticulin, SNARE-like, bHLH, and MADS-box—generally showed their highest expression or a marked increase at IS11 (Figure 5C), consistent with the broad C2 trajectory during post-initiation elongation.

Because *StLAX5* had only weak membership across the six Mfuzz clusters, its temporal profile was examined separately in view of its reported role in lateral stolon primordium formation [14] (Supplementary Figure S4). Mean *StLAX5* transcript abundance was similar at IS0 and IS5 (41.06 and 40.86 TPM), declined at IS7 and IS9 (17.79 and 14.96 TPM), and partially recovered at IS11 (29.58 TPM). *StLAX5* was downregulated in IS7 vs. IS5 (log_2_FC = −1.294, adjusted *p* = 2.36 × 10^−8^) and upregulated in IS11 vs. IS9 (log_2_FC = 0.987, adjusted *p* = 1.01 × 10^−9^); it was not classified as a DEG in IS5 vs. IS0 or IS9 vs. IS7. Its maximum Mfuzz membership was 0.230 in C1, indicating that the profile did not closely match any of the six broad trajectories.

### 2.6. Comparison of RT-qPCR and RNA-Seq Expression Profiles

**Figure 6:**
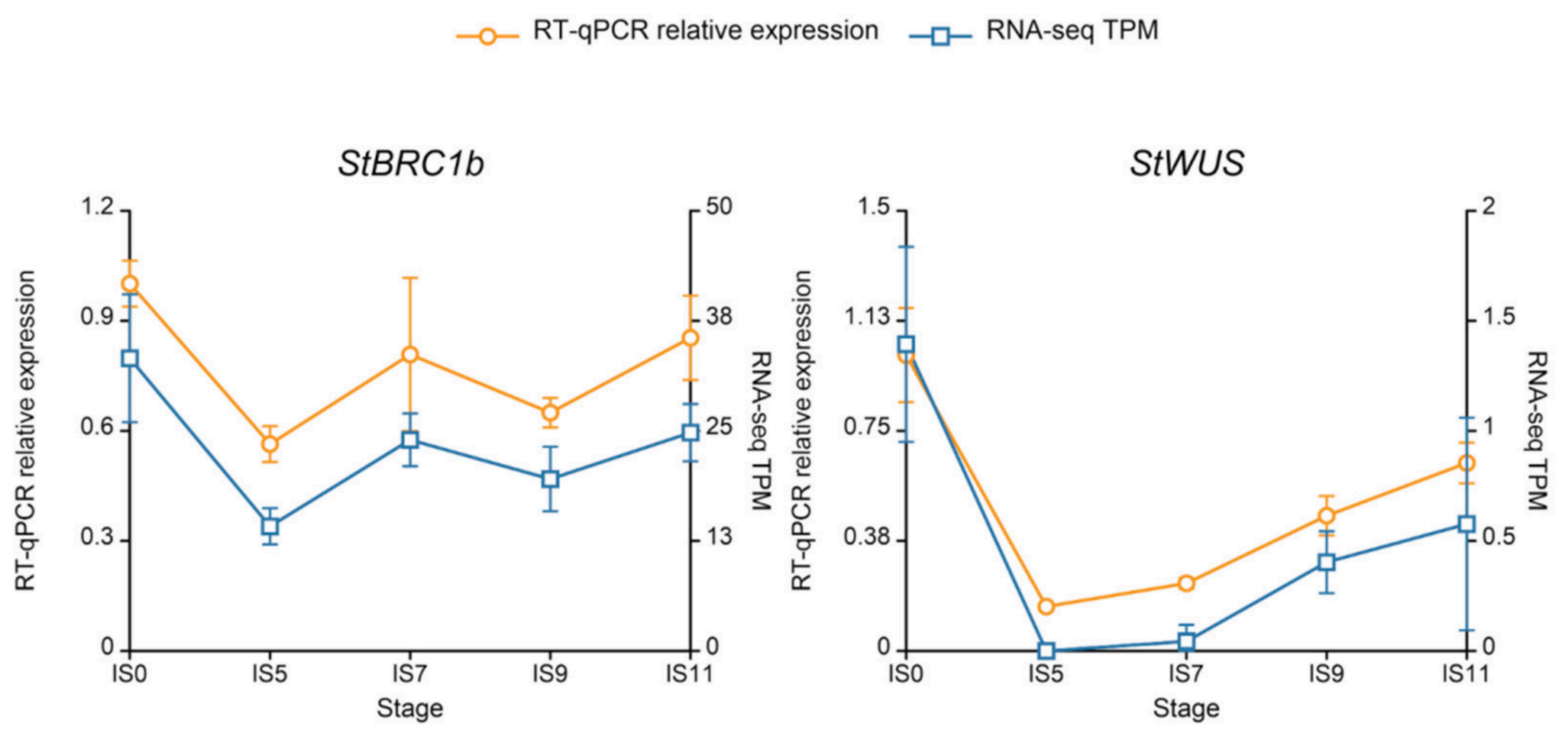

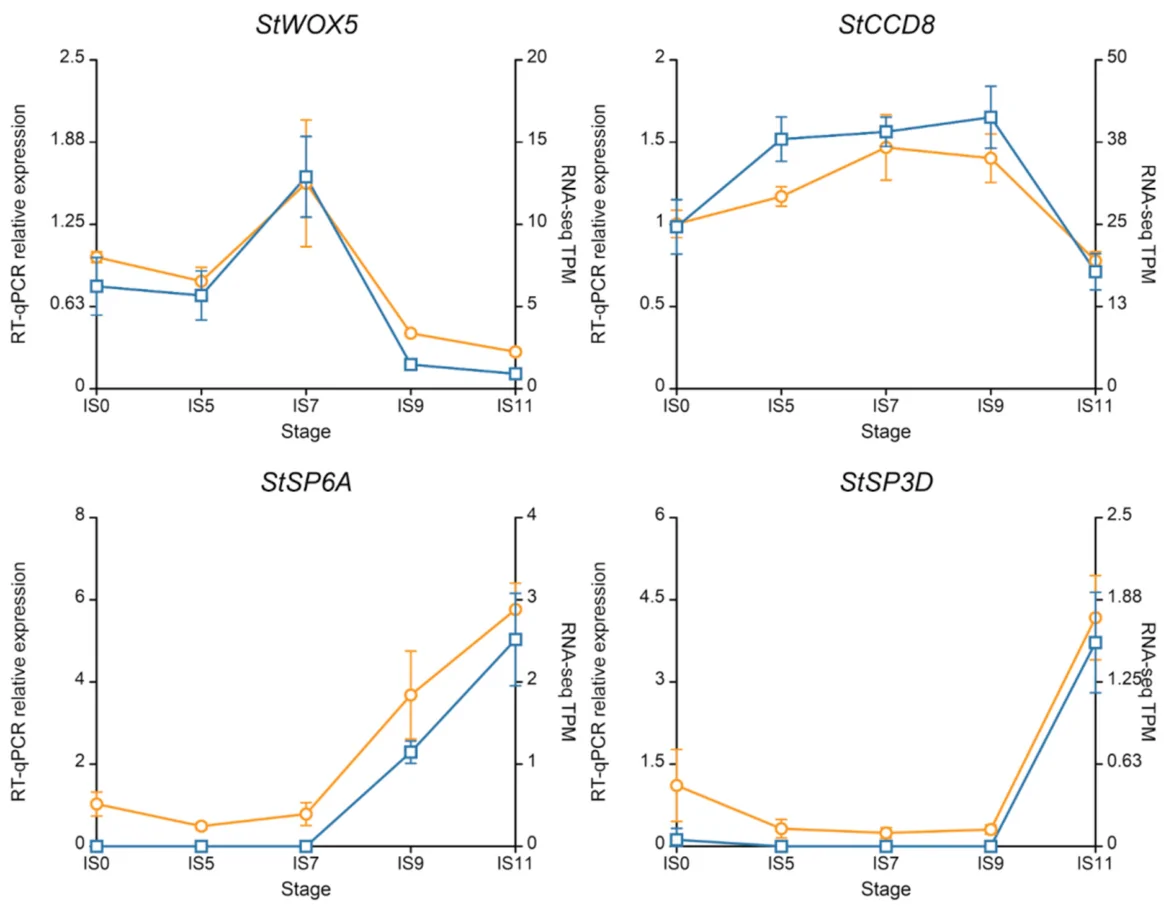
Comparison of RT-qPCR and RNA-Seq expression profiles of selected genes across developmental stages. Temporal profiles of *StBRC1b* (Soltu.DM.06G025210.1), *StWUS* (Soltu.DM.02G023940.1), *StWOX5* (Soltu.DM.03G012800.1), *StCCD8* (Soltu.DM.01G033760.1), *StSP6A* (Soltu.DM.05G026370.1), and *StSP3D* (Soltu.DM.03G011110.1) at IS0, IS5, IS7, IS9, and IS11. Orange circles and lines show relative expression measured by RT-qPCR and correspond to the left *y*-axis. Relative expression was calculated using the 2^−ΔΔCq^ method, with *StACTIN* as the internal reference gene and IS0 as the calibrator for each target. Blue squares and lines show RNA-Seq expression in transcripts per million (TPM) and correspond to the right *y*-axis. The independent axes preclude direct comparison of absolute values. Data are presented as the mean ± SD of three biological replicates for each dataset.

Temporal expression profiles of *StBRC1b*, *StWUS*, *StWOX5*, *StCCD8*, *StSP6A*, and *StSP3D* were compared using RT-qPCR relative expression and RNA-Seq TPM values (Figure 6). The datasets were plotted on independent y-axes because their absolute values are not directly comparable.

*StBRC1b* decreased from IS0 to IS5, increased at IS7, declined moderately at IS9, and increased again at IS11 in both datasets. *StWUS* was highest at IS0, remained low at IS5 and IS7, and increased at IS9 and IS11. *StWOX5* reached a distinct maximum at IS7 and declined markedly thereafter. *StCCD8* increased after IS0, remained comparatively high during IS5–IS9, and decreased at IS11. *StSP6A* was low during the earlier stages and increased at IS9 and IS11, whereas *StSP3D* showed a pronounced increase at IS11. Minor differences occurred at some low-expression stages, particularly for *StSP6A* and *StSP3D*.

The RT-qPCR profiles were broadly consistent with the principal temporal trends observed by RNA-Seq.

## 3. Discussion

Potato subterranean development is commonly interpreted through the framework of photoperiod-dependent tuber induction and the subsequent stolon-to-tuber transition. This framework has established the importance of FT-like signals, the tuberigen complex, source–sink regulation, and transcriptional regulators such as StSP6A, StSP5G, StCOL1, StBEL5, StSP3D, and StFTL1 [1,2,3,4,5,6,7,8,9,10]. However, these mechanisms principally concern established stolons, stolon-tip swelling, or tuber formation. The present study addresses an earlier developmental interval: the transition of an initially appressed basal axillary bud into a morphologically established subterranean lateral axis. The absence of visible stolon-tip swelling or storage-tissue differentiation at IS11 confirms that the series ends during post-initiation elongation rather than tuberization. In this respect, the present time course complements recent transcriptomic analyses of the stolon-to-tuber transition, single-cell comparisons of shoot apices and established stolon tips, sucrose-induced stolon initiation, and functional analysis of individual stolon-initiation regulators [14,22,24,25].

The IS5 stage reveals that the response to soil covering began before visible bud reorientation. Although bud angle and length remained similar to those at IS0, tissue organization had already changed in the apical region and at the bud–main stem junction. The accompanying transcriptional pattern was not consistent with a simple, generalized acceleration of growth. Genes upregulated in IS5 versus IS0 were enriched in oxidative-stress, hydrogen-peroxide-catabolism, defense, and phenylpropanoid-related categories, whereas cell-division, microtubule, DNA-replication, and homologous-recombination categories were enriched among downregulated genes. The transient IS5 maximum of C6 provides a broader temporal context for this transition, but the adjacent-stage analysis more directly shows that early tissue reorganization involved a change in developmental and environmental-response state rather than uniform activation of cell proliferation.

Soil covering alters several features of the basal-node environment simultaneously, including light exposure, gas diffusion, moisture, and mechanical contact. The transient expression of AP2/ERF-, ADH-, and PDC-annotated genes is compatible with an oxygen-responsive transcriptional signature, although it does not establish that hypoxia occurred [26]. The presence of SAUR-like and GH3 genes and the enrichment of auxin-response annotations indicate that auxin-responsive transcription also formed part of the early response. This interpretation is consistent with the broader role of auxin in axillary-bud development and with genetic evidence that the auxin influx carrier *StLAX5* contributes to potato stolon primordium formation [14,15,16]. Nevertheless, *StLAX5* itself was not transcriptionally induced during IS7–IS9 in the present bulk series. Its comparatively high abundance at IS0–IS5 therefore indicates that the functional contribution established genetically is not represented by a simple initiation-window increase in whole-sample transcript abundance.

The two transitions within the morphologically evident initiation interval were also distinct. From IS5 to IS7, the first clear separation of the bud from the main stem was accompanied by enrichment of amino-acid-transport, sulfur-metabolism, and pentose-phosphate-pathway categories among upregulated genes and by reduced representation of photosynthesis-, light-harvesting-, and porphyrin-metabolism-related transcripts. This pattern is consistent with a shift from the physiology of an exposed axillary bud toward that of a covered, increasingly heterotrophic lateral axis. Carbon availability and hormone–carbon interactions are known to influence axillary-bud outgrowth in branching systems [15,16,19], and sucrose-induced potato stolon initiation is likewise associated with broad hormonal and metabolic transcriptional changes [25]. The soil-covering and sucrose-induction systems are not developmentally identical, but both indicate that the transition to stolon growth involves more than a localized change in meristem identity and includes adjustment of carbon and intermediary metabolism.

The IS7-to-IS9 transition showed a different annotation profile. Glutathione metabolism, flavonoid biosynthesis, and phenylpropanoid biosynthesis were enriched among upregulated genes, whereas cytoskeletal, cell-cycle, steroid-biosynthesis, and hormone-signaling categories were represented among downregulated genes. Thus, the visible increase in basal enlargement and lateral-axis extension was not accompanied by a uniform increase in all growth-associated categories. Instead, the data suggest sequential reorganization of redox-related, specialized-metabolic, structural, and hormonal transcription. This temporal subdivision is also reflected in the distinct C3 and C5 trajectories: C3 began to increase at IS5 and reached its maximum at IS9, whereas C5 increased mainly during IS7–IS9 and declined sharply at IS11. The initiation interval should therefore not be treated as a transcriptionally synchronous state.

Comparable temporal complexity has been reported in other lateral-bud systems. In woodland strawberry, light, gibberellin, endogenous developmental competence, and *ZFP6* contribute to the choice between branch-crown and stolon development [17,18]. In petunia, ABA-associated transcription and carbon availability distinguish buds with different outgrowth behavior [19]. These systems are not direct models of potato subterranean development, but they support the broader interpretation that lateral-axis establishment emerges from coordinated hormonal, metabolic, and developmental inputs rather than from a single stage-specific switch. The present results extend this concept to soil-covered potato basal buds by resolving an early internal response from subsequent visible axis establishment.

The enrichment of protein-processing and SNARE-mediated-transport annotations in the combined C3 and C5 gene set provides an additional cellular context for the initiation interval. Formation of a new lateral axis requires coordinated changes in membrane trafficking, secretion, plasma-membrane composition, and cell-wall organization. In *Arabidopsis*, post-Golgi trafficking and clathrin-mediated endocytosis jointly regulate plasma-membrane cargo dynamics [27], while SYP132-dependent secretion contributes to the delivery of extracellular and cell-wall-associated cargoes [28]. Cell-wall synthesis and modification are closely integrated with cytoskeletal and membrane-trafficking systems [29], and disruption of ESCRT-dependent multivesicular endosome formation affects normal plant growth [30]. The enrichment observed here does not demonstrate increased trafficking activity, but its temporal coincidence with basal enlargement and lateral-axis establishment is consistent with membrane and cell-wall remodeling during stolon initiation.

IS11 represented a second major transcriptional transition, but its biological meaning differs from stolon-tip swelling. Translation-, ribosome-, proteasome-, nucleotide-sugar-, microtubule-, and cell-division-related categories were enriched among upregulated genes in IS11 versus IS9, and C2 was associated with translation, energy metabolism, protein processing, and intracellular transport. These annotations are consistent with the biosynthetic and structural requirements of sustained longitudinal growth after the stolon axis had formed. The later increase in *StSP6A* and *StSP3D* is noteworthy because FT-like proteins and tuberigen-complex components participate in long-distance signaling and storage-organ development [5,6,10,31]. Their accumulation, however, does not by itself classify IS11 as tuberization. Functional analysis of *StbHLH93* has associated proplastid-to-amyloplast differentiation with stolon swelling [32], whereas the IS11 samples showed neither visible tip swelling nor storage-tissue differentiation. The morphology therefore places IS11 before the stolon-to-tuber transition, even though some transcripts associated with later subterranean development had begun to increase.

The selected genes also differ in the strength of existing functional evidence. *StBRC1b* contributes to the spatial control of potato subterranean development, and *StCCD8*-dependent strigolactone biosynthesis affects branching and stolon architecture [11,12,13]. *StLAX5* has direct genetic support for a role in stolon primordium formation [14], although its expression in the present time series did not follow a simple IS7–IS9 induction pattern. By contrast, no direct role in potato stolon initiation has been established for *StWUS* or *StWOX5*. Their temporal profiles are relevant because WUSCHEL-related homeobox proteins regulate meristem organization and stem-cell maintenance in other developmental contexts [20,21], but they remain candidates rather than validated regulators. The broad agreement between RT-qPCR and RNA-Seq supports the reliability of their temporal profiles, not their causal involvement in the transition.

**Figure 7:**
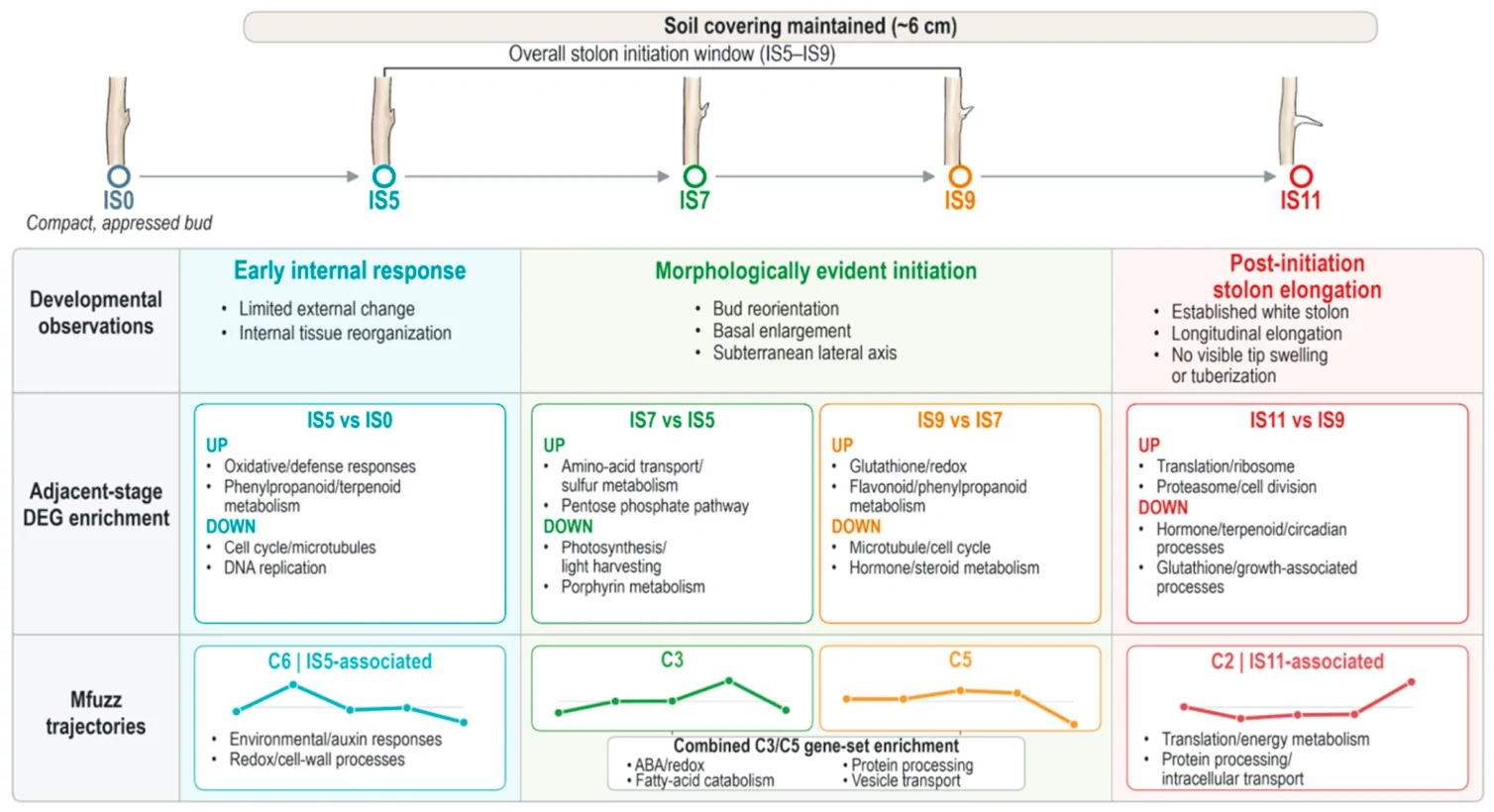
Integrative working model of soil-covering-induced potato stolon initiation. Soil covering was applied immediately after IS0 and maintained at approximately 6 cm through IS11. IS5–IS9 constitutes the overall initiation window: IS5 represents an early internal response, IS7–IS9 represents morphologically evident stolon initiation and subterranean lateral-axis establishment, and IS11 represents post-initiation elongation without visible tip swelling or tuberization. The middle row summarizes the principal significantly enriched categories among upregulated and downregulated DEG sets in the four adjacent-stage comparisons. UP and DOWN refer to expression changes at the later stage relative to the earlier stage. The bottom row shows the C6, C3, C5, and C2 Mfuzz trajectories; C3 and C5 remained separate clusters and were combined only for one enrichment gene set. Arrows denote developmental progression rather than regulatory direction.

Therefore, this study presents an evidence-aligned developmental model. Bulk RNA-Seq integrates signals from the bud apex, bud–main stem junction, developing stolon, and adjacent tissues, whereas single-cell analysis has shown substantial cellular heterogeneity between shoot apices and stolon tips [24]. Spatial expression analysis and targeted genetic studies will be required to determine where the prioritized genes act and whether the associated processes are necessary for stolon initiation. Nevertheless, integrating morphology with adjacent-stage enrichment and broader temporal trajectories distinguishes an early internal response, a temporally structured initiation interval, and post-initiation elongation, thereby extending potato subterranean-development research upstream of the conventional stolon-to-tuber framework. These stage-specific relationships are summarized in the integrative working model shown in Figure 7.

## 4. Materials and Methods

### 4.1. Plant Materials, Soil-Covering Treatment, and Sampling

Tissue-cultured plantlets of potato (*Solanum tuberosum* L. cv. Désirée) were maintained and propagated at the School of Agriculture, Yunnan University. One month after subculture on Murashige and Skoog medium (Solarbio Science & Technology Co., Ltd., Beijing, China), plantlets were transferred to pots containing humus soil, vermiculite, and perlite at a ratio of 6:2:1 (*v*/*v*/*v*) and grown in a greenhouse at 22 °C under a 14-h light/10-h dark photoperiod. Plants were watered as needed and supplied every 3 d with a commercial water-soluble compound fertilizer (Huawuque, Shanghai Wintong Ecological Engineering Co., Ltd., Shanghai, China; N-P_2_O_5_-K_2_O = 20-20-20, supplemented with trace elements) at a concentration of 20 g L^−1^. A representative culture vessel and plantlet are shown in Supplementary Figure S1.

Uniformly growing plants with visible basal axillary buds but no pronounced lateral elongation were selected. Soil was added around the basal stem to a depth of approximately 6 cm, covering the basal nodes and associated axillary buds up to the fourth node from the base. Samples collected immediately before soil covering were designated IS0; samples collected 5, 7, 9, and 11 d after treatment were designated IS5, IS7, IS9, and IS11, respectively. The prefix “IS” identifies this developmental series, and the numeral indicates days after soil covering.

At each stage, tissue was collected from the same predefined basal axillary-bud position on each independent plant. Each bulk sample included the bud apex, the bud–stem junction, stage-dependent bud or stolon tissue, and a standardized length of adjacent main-stem tissue. The stage-dependent component comprised the intact basal axillary bud at IS0 and IS5, the developing lateral axis at IS7 and IS9, and the proximal region of the established white subterranean stolon at IS11. Thus, nodal position, junctional region, and adjacent main-stem boundaries were standardized despite the changing morphology of the bud-derived tissue. RNA-Seq and RT-qPCR used the same stage definitions and sampling boundaries. Three biological replicates were prepared per stage, each consisting of an equal total mass of tissue pooled from 15 independent plants. Samples were frozen immediately in liquid nitrogen and stored at −80 °C. Six independent, unpooled plants per stage were used for morphometric measurements.

### 4.2. Morphological and Histological Analyses

Morphological and histological samples were collected from the predefined basal axillary-bud position using the standardized main-stem boundaries described in Section 4.1. Basal axillary buds and developing subterranean stolons were photographed at IS0, IS5, IS7, IS9, and IS11. Bud orientation, separation from the main stem, basal enlargement, lateral extension, and white-stolon formation were recorded. Digital images were calibrated and analyzed using ImageJ v1.54t (National Institutes of Health, Bethesda, MD, USA). The angle between the basal axillary bud or developing stolon axis and the main stem and the length of the bud or developing stolon were measured in six independent plants per stage. For histological analysis, samples were fixed for 24 h at room temperature in FAA solution containing 70% ethanol, 37% formaldehyde solution, and glacial acetic acid (all from Sinopharm Chemical Reagent Co., Ltd., Shanghai, China) at 90:5:5 (*v*/*v*/*v*), dehydrated through a graded ethanol series, cleared in xylene (Sinopharm Chemical Reagent Co., Ltd., Shanghai, China), and embedded in paraffin (Leica Biosystems Nussloch GmbH, Nussloch, Germany). Longitudinal sections were prepared along the bud or stolon axis using a Leica RM2235 rotary microtome (Leica Biosystems Nussloch GmbH, Nussloch, Germany), cut at 8 μm, stained with 0.05% toluidine blue O (Solarbio Science & Technology Co., Ltd., Beijing, China), mounted in neutral balsam (Solarbio Science & Technology Co., Ltd., Beijing, China), and examined using an Olympus BX53 light microscope (Olympus Corporation, Tokyo, Japan). Observations focused on the bud apical region, bud–main stem junction, basal tissue organization, and longitudinal extension of the developing lateral axis.

### 4.3. RNA Extraction, Library Construction, and Sequencing

Total RNA was extracted using the RNAprep Pure Plant Plus Kit (TIANGEN Biotech (Beijing) Co., Ltd., Beijing, China) according to the manufacturer’s instructions, and residual genomic DNA was removed using RNase-free DNase I (TIANGEN Biotech (Beijing) Co., Ltd., Beijing, China). RNA concentration and purity were measured using a NanoDrop 2000 spectrophotometer (Thermo Fisher Scientific, Wilmington, DE, USA), and RNA integrity was assessed using an Agilent 2100 Bioanalyzer (Agilent Technologies, Santa Clara, CA, USA). Samples with an A260/A280 ratio of 1.8–2.1 and an RNA integrity number ≥ 7.0 were used for library construction. Approximately 1 μg of total RNA from each biological replicate was used, and poly(A)-containing mRNA was enriched with oligo(dT) magnetic beads (BGI Genomics, Shenzhen, China). Libraries were prepared and sequenced by BGI Genomics (Shenzhen, China) on a DNBSEQ-T7 platform (MGI Tech Co., Ltd., Shenzhen, China) to generate 150-bp paired-end reads. Fifteen libraries were generated, representing five developmental stages with three biological replicates per stage.

### 4.4. RNA-Seq Processing and Differential Expression Analysis

Raw reads were processed using fastp v0.23.4 to remove adapter sequences, reads containing excessive undetermined bases, and low-quality reads [33]. Clean reads were aligned to the *Solanum tuberosum* DM v6.1 reference genome [34] using HISAT2 v2.2.1 [35], and gene-level raw read counts were generated using featureCounts v2.0.3 with the corresponding DM v6.1 annotation [36]. Gene expression levels were expressed as transcripts per million (TPM); TPM values were used for expression visualization, heatmaps, representative-gene profiles, and temporal clustering, whereas raw counts were used for differential expression. Principal component analysis used a variance-stabilized expression matrix generated with DESeq2 v1.42.0 [37].

Differential expression analysis was conducted with DESeq2 using raw counts as input [37], and *p* values were adjusted by the Benjamini–Hochberg procedure [38]. Genes with adjusted *p* < 0.05 and non-zero log_2_ fold change were classified as DEGs; positive and negative log_2_ fold changes denoted upregulation and downregulation at the later stage, respectively. No additional fold-change magnitude threshold was applied. Adjacent-stage comparisons were IS5 vs. IS0, IS7 vs. IS5, IS9 vs. IS7, and IS11 vs. IS9. Separate IS0-reference comparisons—IS5 vs. IS0, IS7 vs. IS0, IS9 vs. IS0, and IS11 vs. IS0—were used for the four-set Venn analyses in Figure 2C,D. Complete gene-level differential-expression results for the adjacent-stage and IS0-reference comparisons are provided in Supplementary Table S2. The stage-wise gene-level *StLAX5* profile in Supplementary Figure S4 was extracted from the same TPM matrix, and the adjacent-stage log_2_FC and adjusted *p* values were taken from the same DESeq2 analysis; no separate normalization or statistical pipeline was applied.

### 4.5. Adjacent-Stage DEG Functional Enrichment Analysis

Direction-specific DEG sets from the four adjacent-stage comparisons were analyzed separately. Genes with Benjamini–Hochberg-adjusted DESeq2 *p* < 0.05 and nonzero log_2_ fold change were retained; positive values defined upregulated DEGs and negative values defined downregulated DEGs at the later stage. GO BP and KEGG term membership, category-level background counts, and total annotated background counts were obtained from the corresponding DM v6.1 annotation-based enrichment tables. For each comparison, direction, and database, the directional DEG set was intersected with the category-membership lists. Let *k* denote the number of directional DEGs assigned to a category, *n* the number of directional DEGs annotated in the relevant database, *K* the number of background genes assigned to the category, and *M* the total annotated background. Over-representation was evaluated using the upper tail of a one-sided hypergeometric distribution, *P*(*X* ≥ *k*), with parameters *M*, *K*, and *n*. The annotated backgrounds contained 15,458 genes for GO BP and 11,993 genes for KEGG. Categories represented by at least one directional DEG (*k* ≥ 1) were tested. *p* values were adjusted independently within each comparison × direction × database combination using the Benjamini–Hochberg procedure, and *Q* < 0.05 was considered significant. Rich Ratio was calculated as *k*/*K*. Complete tested results, including non-significant categories, are provided in Supplementary Tables S3 and S4.

GO BP and KEGG results were processed separately for Figure 3. Within each of the eight comparison-by-direction combinations, significant categories were ranked by ascending *Q* value, descending Rich Ratio, and then stable identifier; up to three categories were selected. The union of the selected categories defined the matrix rows, and all significant occurrences across the eight combinations were displayed. Bubble area represents Rich Ratio, and bubble color represents −log_10_(*Q* value), capped at 6 for display. The figure was generated in R v4.5.1 (R Foundation for Statistical Computing, Vienna, Austria) using ggplot2 v4.0.0 and patchwork v1.3.2.

### 4.6. Temporal Clustering, Cluster-Based Enrichment, and Transcription Factor Annotation

Genes with TPM ≥ 1 in at least three of the 15 libraries were retained for temporal analysis. TPM values were averaged across the three biological replicates at each stage, transformed using log_2_(TPM + 1), and converted to gene-wise z-scores across stages. Temporal soft clustering was performed in R v4.3.2 (R Foundation for Statistical Computing, Vienna, Austria) using Mfuzz v2.62.0 [39], with fuzzifier *m* = 1.25. Six clusters were used to provide an interpretable summary of broad expression trajectories. The solution was treated as descriptive rather than as a uniquely optimized partition of developmental states. Each gene received a membership value, and membership ≥ 0.7 defined high-membership trajectories for visualization in Figure 4. This threshold did not restrict the gene sets used for cluster-based enrichment.

GO BP and KEGG enrichment analyses were performed with clusterProfiler v4.10.1 [40] for all genes assigned to C6, all genes assigned to C2, and the union of genes assigned separately to C3 and C5. C3 and C5 remained distinct Mfuzz clusters and were combined only at the enrichment gene-set level. Gene-to-term relationships were derived from the DM v6.1 functional annotation, Gene Ontology [41], and KEGG [42]; all genes retained for temporal clustering with relevant annotations constituted the background. *p* values were adjusted using the Benjamini–Hochberg procedure, *Q* < 0.05 was considered significant, and the 20 categories with the lowest *Q* values were displayed in Supplementary Figure S2. Complete results are provided in Supplementary Tables S6 and S7.

C1 and C4 were analyzed separately with the same clusterProfiler v4.10.1 workflow, DM v6.1 GO and KEGG annotations, annotated temporal-clustering background, Benjamini–Hochberg correction, and *Q* < 0.05 threshold. Only significant categories are displayed in Supplementary Figure S3; when more than 20 significant categories were identified, the 20 categories with the lowest *Q* values are shown.

Transcription factors were assigned to families according to PlantTFDB v5.0 (Gao Lab, Peking University, Beijing, China) family rules and conserved DNA-binding-domain architecture. Supplementary Table S8 lists transcription factors with membership ≥ 0.7 in C2, C3, C5, and C6. Representative genes displayed in Figure 5 were selected according to cluster assignment, temporal trajectory, membership value, functional annotation, correspondence with enrichment results, and nonredundant functional coverage; selection was not restricted to high-membership genes.

### 4.7. RT-qPCR Analysis

Six genes were analyzed by RT-qPCR: *StBRC1b* (Soltu.DM.06G025210.1), *StWUS* (Soltu.DM.02G023940.1), *StWOX5* (Soltu.DM.03G012800.1), *StCCD8* (Soltu.DM.01G033760.1), *StSP6A* (Soltu.DM.05G026370.1), and *StSP3D* (Soltu.DM.03G011110.1). First-strand cDNA was synthesized from 1 μg of total RNA using the PrimeScript RT Reagent Kit with gDNA Eraser (Takara Bio Inc., Kusatsu, Shiga, Japan). RT-qPCR was performed in 20 μL reactions using TB Green Premix Ex Taq II (Takara Bio Inc., Kusatsu, Shiga, Japan) on a Bio-Rad CFX96 Real-Time PCR Detection System (Bio-Rad Laboratories, Inc., Hercules, CA, USA). The program comprised 95 °C for 30 s; 40 cycles of 95 °C for 5 s and 60 °C for 30 s; and melting-curve analysis. Three biological replicates were analyzed per stage, each comprising pooled tissue from 15 independent plants, and each biological sample was measured in three technical reactions. Technical Cq values were averaged before relative expression was calculated. *StACTIN* was the internal reference gene, with primers 5′-CAAGTTATTACCATTGGTGCTGAGA-3′ and 5′-TGCAGCTTCCATACCAATCATG-3′. Relative expression was calculated using the 2^−ΔΔCq^ method [43], with IS0 as the calibrator for each target, and is presented as the mean ± SD of three biological replicates. Target-gene primer sequences, transcript identifiers, and amplicon information are provided in Supplementary Table S10. RT-qPCR terminology follows MIQE 2.0 recommendations [44].

### 4.8. Statistical Analysis

Morphometric and RT-qPCR data are presented as the mean ± SD. Morphometric measurements from six independent plants per stage were analyzed by one-way analysis of variance followed by Tukey’s multiple-comparison test using GraphPad Prism 8.0.2 (GraphPad Software, San Diego, CA, USA). Differences were considered significant at *p* < 0.05, and different lowercase letters indicate statistically distinct groups. RT-qPCR data were summarized descriptively from three biological replicates per stage for comparison with RNA-Seq temporal trends; technical reactions were averaged and were not treated as independent biological observations.

## 5. Conclusions

By integrating morphological, histological, and transcriptomic evidence, this study resolved the transition from a basal axillary bud to a subterranean stolon into an IS5 early internal response, morphologically evident initiation during IS7–IS9, and post-initiation elongation at IS11. Adjacent-stage differential expression and direction-resolved enrichment distinguished the successive transitions, including early oxidative/defense and phenylpropanoid-related responses, intermediate changes in transport, photosynthesis, redox metabolism, and cytoskeletal organization, and the later increase in translation, proteasome, and cell-division functions during stolon elongation. Mfuzz clustering provided a complementary overview of broader temporal trajectories, with C6 associated with IS5, distinct C3 and C5 patterns across the initiation interval, and C2 increasing at IS11. RT-qPCR profiles were broadly consistent with the RNA-Seq trends of the selected genes. Together, these findings define a stage-resolved framework that separates stolon initiation from subsequent elongation and later tuberization.

## Data Availability

The raw RNA-Seq reads generated in this study have been deposited in the NCBI Sequence Read Archive (SRA) under BioProject accession PRJNA1501893. Processed DEG results, Mfuzz cluster assignments and membership values, GO and KEGG enrichment results, transcription factor annotations, primer sequences, and other supporting datasets are provided in the Supplementary Materials.

## Supplementary Materials

The following supporting information can be downloaded at: https://www.mdpi.com/article/10.3390/ijms27177633/s1.
