# Supplementary material for: Time-Resolved Developmental and Transcriptomic Profiling of Potato Stolon Initiation from Basal Axillary Buds Following Soil Covering
Source: Int J Mol Sci. 2026 Aug 26;27(17):7633. doi: 10.3390/ijms27177633 (PMC13565819; doi:10.3390/ijms27177633)
Supplement: Supplementary file 1 [file ijms-27-07633-s001.zip › 05_Supplementary_Materials.pdf]

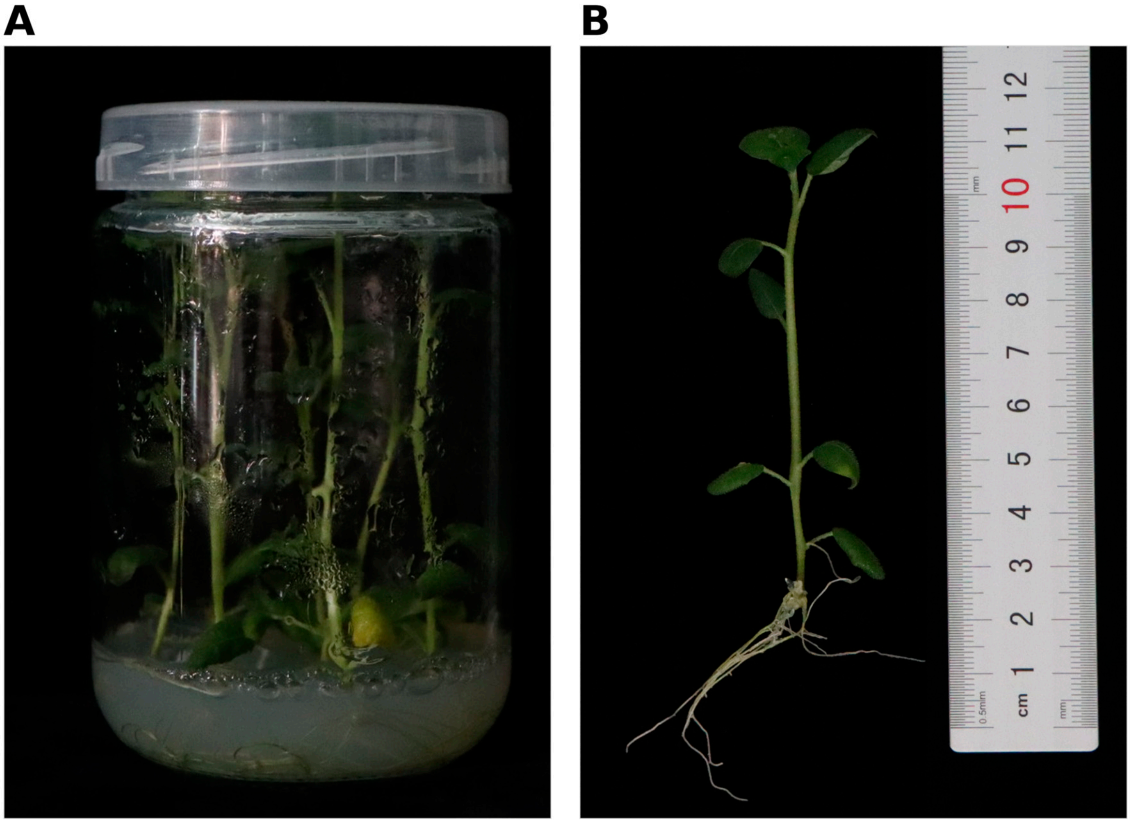

Figure S1. Tissue-cultured potato cv. Désirée plantlets used for propagation. (A) Plantlets maintained on Murashige and Skoog medium before transfer to pots. (B) Representative one-month-old plantlet removed from culture and shown beside a ruler for scale.

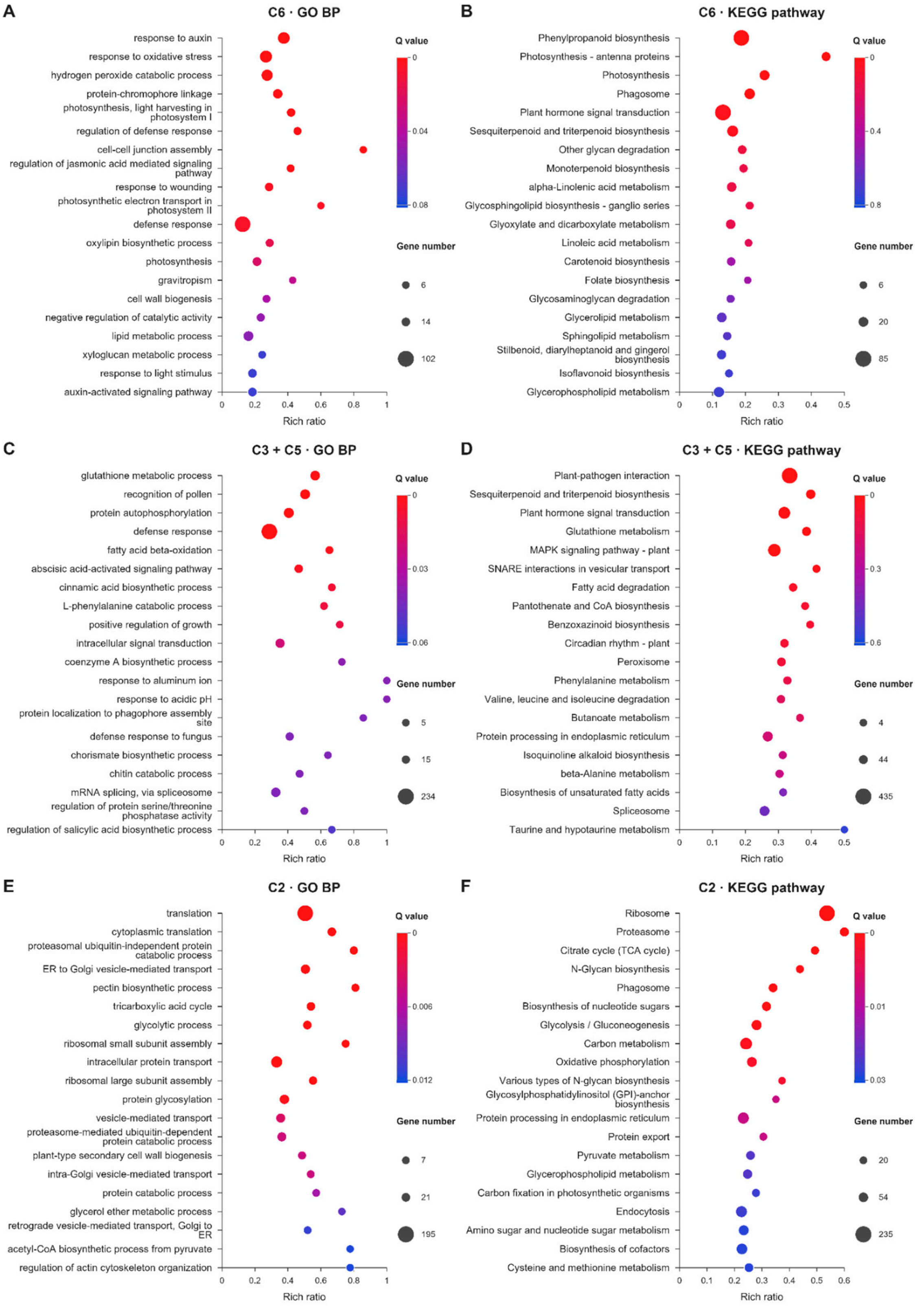

Figure S2. Cluster-based functional enrichment profiles for broad temporal trajectories. (A,B) GO Biological Process terms and KEGG pathways for all genes assigned to C6. (C,D) Results for the union of all genes assigned separately to C3 and C5; the two clusters remained distinct and were combined only at the enrichment gene-set level. (E,F) Results for all genes assigned to C2. Each panel shows the 20 categories with the lowest Q values; Q < 0.05 was considered significant. Rich Ratio is shown on the x-axis, point size indicates the number of input genes assigned to the category, and color indicates Q value. Complete results are provided in Supplementary Tables S4 and S5.

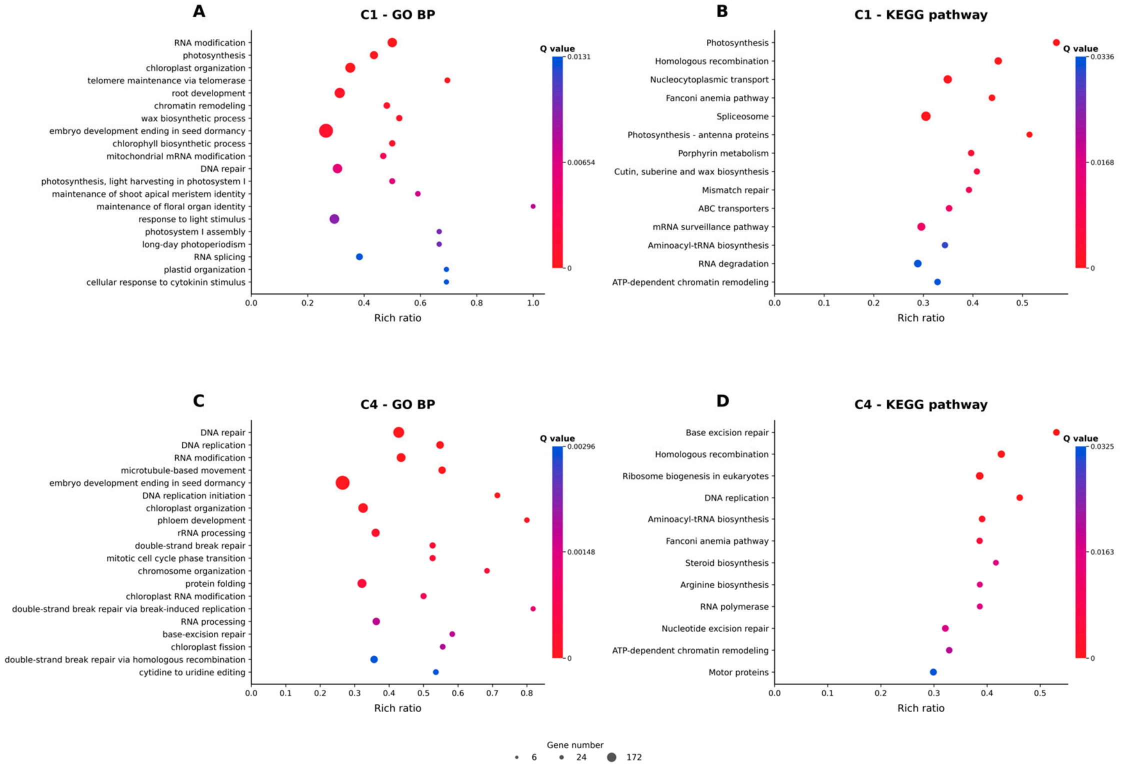

Figure S3. GO Biological Process and KEGG enrichment profiles for C1 and C4. (A,B) GO BP terms and KEGG pathways for all genes assigned to C1 by maximum Mfuzz membership. (C,D) Corresponding results for all genes assigned to C4. C1 and C4 were analyzed separately using the same clusterProfiler v4.10.1 workflow, DM v6.1 annotations, annotated temporal-clustering background, Benjamini–Hochberg correction, and Q < 0.05 threshold used for the selected temporal clusters. Only significant categories are displayed; when more than 20 significant categories were identified, the 20 categories with the lowest Q values are shown. Rich Ratio is shown on the x-axis, point size indicates gene number, and color indicates Q value.

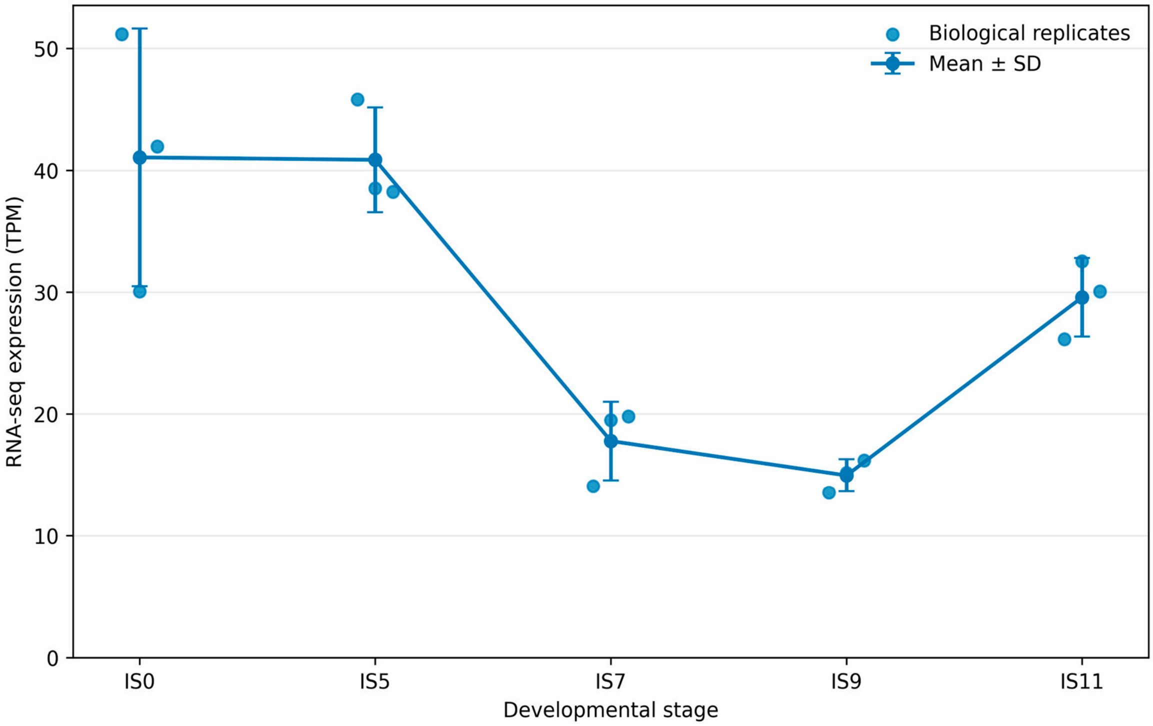

Figure S4. Temporal RNA-seq expression profile of *StLAX5* (Soltu.DM.11G010760) across IS0, IS5, IS7, IS9, and IS11. Individual points show the three biological replicates; the line and error bars show the mean ± SD TPM. Mean TPM values were 41.06, 40.86, 17.79, 14.96, and 29.58, respectively. In adjacent-stage DESeq2 comparisons, *StLAX5* was downregulated in IS7 vs. IS5 (log_2FC = −1.294, adjusted P = 2.36 × 10−8) and upregulated in IS11 vs. IS9 (log_2FC = 0.987, adjusted P = 1.01 × 10−9); it was not classified as a DEG in IS5 vs. IS0 or IS9 vs. IS7. Its maximum Mfuzz membership was 0.230 in C1, indicating weak conformity to any single broad trajectory.
